# Imaging of formaldehyde in plants with a ratiometric fluorescent probe†
†Electronic supplementary information (ESI) available. See DOI: 10.1039/c7sc00373k



**DOI:** 10.1039/c7sc00373k

**Published:** 2017-06-06

**Authors:** Zhen Li, Yuqing Xu, Hailiang Zhu, Yong Qian

**Affiliations:** a State Key Laboratory of Pharmaceutical Biotechnology , School of Life Sciences , Nanjing University , No. 163 Xianlin Road , Nanjing 210023 , China . Email: yongqian@nju.edu.cn; b College of Chemistry and Materials Science , Nanjing Normal University , No. 1 Wenyuan Road , Nanjing , 210046 , China . Email: yongqian@njnu.edu.cn

## Abstract

The fluorescence monitoring of formaldehyde in real environmental samples and live plant tissues is of great importance for physiological and pathological studies. However, there is a lack of suitable chemical tools to directly trace and measure the formaldehyde activity in bio-systems, and developing effective and, in particular, selective sensors for mapping formaldehyde in live tissues still remains a great challenge. Here, we demonstrate for the first time that the ratiometric fluorescence monitoring of formaldehyde in live plant tissues is achieved with a newly developed ratiometric fluorescent probe, **FAP**, which effectively eliminated interference from other comparative analytes. Live tissue analyses reveal that **FAP** can potentially detect exogenous and endogenous formaldehyde in live *Arabidopsis thaliana* tissues, exposing a potential application for biological and pathological studies of formaldehyde.

## Abstract 2

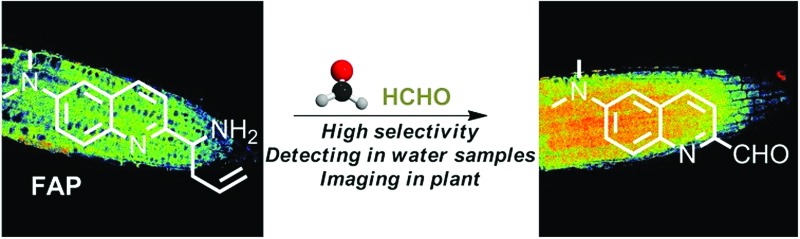
We demonstrate that the ratiometric fluorescence monitoring of formaldehyde in live plant tissues is achieved with a newly developed ratiometric fluorescent probe, **FAP**, which effectively eliminated interference from other comparative analytes.

## Introduction

Formaldehyde (FA), the simplest of the aldehydes with the formula H–CHO, has been widely used as an important precursor to many industrial materials and chemical compounds.^1^ On the other hand, it has also been classified as a highly toxic compound to living organisms, because its strong interactions with proteins, nucleic acids and other biomolecules will lead to the inactivation of their biological activities.^2^ For instance, excess exposure of exogenous FA to the human body will lead to a significant health threat and cause many diseases,^3–6^ including various cancers, heart disorders, diabetes, *etc.* Indeed, it has been regarded as a suspected carcinogen and well-known human toxicant. In plants, FA pollution is a widespread problem that can affect plant growth and development, causing a significant decrease of the productivity and quality in agriculture.^7^ But, interestingly, intracellular FA can be endogenously formed by normal physiological processes and spontaneously removed by complex metabolism using detoxification mechanisms. It has been demonstrated that glutathione-dependent FA dehydrogenase (FALDH), as a key enzyme of FA metabolism, plays an essential role in the uptake and detoxification of FA in *Arabidopsis* plants.^8^ Thus, the concentration of FA in living organisms is generally maintained in a dynamic state of equilibrium ranging from low to high millimolar levels.^8–11^ For instance, a small pool of FA (0.1–10 μmol g^–1^ fresh weight) was identified in the intermediate of the C1 metabolism of plants.^12,13^ The primary sources of FA in plants are caused by the processes of methanol oxidation, dissociation of 5,10-methylene-THF, glyoxylate decarboxylation and other oxidative demethylation reactions.^14^ Exogenous FA can also be absorbed and incorporated into the metabolism of plants.^7,15,16^ As a result, phytoremediation has been extensively applied for the removal of FA from the polluted environment. However, the biological roles of FA and the mechanism of FA metabolism in plants have not yet been clearly defined,^14^ and they are still challenging to study due to the lack of tools that can map the distribution of FA in live tissues. Thus, in order to understand the biological and pathological roles of FA well, it is crucial to develop sensitive methods or chemical tools for tracing and identifying FA in environmental samples and live plant tissues.

**Scheme 1:**
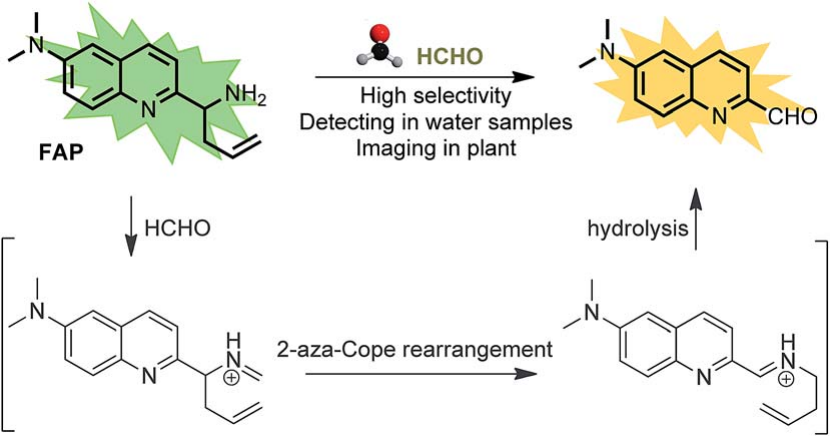
The structure of **FAP** and the proposed ratiometric sensing mechanism for monitoring formaldehyde (FA).

Coupled with fluorescence microscopy techniques, fluorescent imaging using small molecular fluorescent probes to study biological species in living bio-systems has attracted great interest owing to its high spatiotemporal resolution, non-invasiveness, high sensitivity, and excellent selectivity,^17–28^ which can offer the opportunity to monitor FA directly and easily in different biological contexts. However, the effective and, in particular, selective mapping of FA activity in live tissues with fluorescent probes still remains a great challenge. Recently, only a few fluorescent turn-on probes have been reported for monitoring FA.^17–21,29^ Some could be successfully used for imaging FA in living cells, others were primarily applied for the *in vitro* determination of FA in water. These intensity-based fluorescent probes may have a number of limitations imposed by the fluorescent turn-on response at a single detection window, which means it is difficult to obtain accurate information when testing the FA concentration. Moreover, these turn-on responses can also vary depending on the experimental conditions, including the probe concentration, excitation intensity, temperature, and instrument efficiency.^30^ To minimize most of the aforementioned artificial and environmental factors, an attractive approach for the determination of FA in environmental samples and live tissues is developing ratiometric fluorescent probes, due to their quantitative tracking ability and self-calibration effect.^23,31,32^ So far, there is only one reported ratiometric fluorescent probe for monitoring FA,^33^ but its relatively low excitation in the UV range and its short emission wavelength might easily be overwhelmed by the tissue’s auto-fluorescence background, thereby limiting the probe’s further application in live tissue. In addition, fluorescent probes that have the ability to image inside live plant tissues to provide relevant information on endogenous FA are particularly attractive, but no such probe has been reported to date. Therefore, developing novel ratiometric probes that can be applied for the *in vivo* imaging of FA with highly sensitive and selective responses is critically important and there is strong demand. We herein present the development and application of a FA probe, **FAP** (Scheme 1), a new ratiometric fluorescent sensor that can selectively report formaldehyde in real water samples and enable tracking of the endogenous FA activity in live tissues of *Arabidopsis thaliana* for the first time.

## Results and discussion

**Scheme 2:**
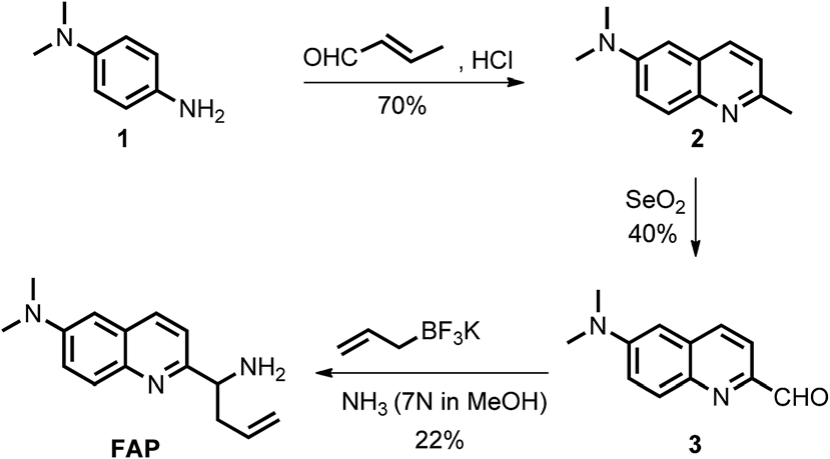
Synthesis of the ratiometric fluorescent probe, **FAP**, for monitoring formaldehyde (FA).

To design this new FA ratiometric fluorescent probe, we focus on the 2-aza-Cope rearrangement reaction,^34^ as illustrated in Scheme 1, where the starting imine intermediate can be formed by the selective capture of FA with a homoallylamino group, and it will further undergo a simple rearrangement and hydrolysis process to yield an aldehyde product,^17,18^ potentially resulting in obvious changes of the fluorescence properties. Thus, we envisioned that the homoallylamino group can possibly serve as the FA selective response site. Subsequently, in order to obtain a ratiometric response, we chose a well-known *N*,*N*-dimethylquinolin-6-amine as the fluorescent chromophore because of its exhibited excellent photophysical and chemical properties,^35,36^ which has been used in the design of other reported ratiometric fluorescent probes as a fluorescence reporter. We anticipated that the free probe would exhibit a relatively short emission wavelength owing to the internal charge transfer (ICT) effect between the *N*,*N*-dimethyl group and the 2-position homoallylamine of the chromophore. Upon treatment with FA, however, the homoallylamino group would change into an aldehyde group *via* specifically activated processes, which would then cause an increase in the electron withdrawing ability, thereby inducing the red shift of the fluorescence emission spectrum of the probe and producing a ratiometric response for FA. Based on these design considerations, we then synthesized the fluorescent probe, **FAP**, in three steps starting from commercially available *N*1,*N*1-dimethylbenzene-1,4-diamine (Scheme 2), and the structures of the target compounds were fully characterized by ^1^H and ^13^C NMR spectroscopy and mass spectrometry (see the ESI†).

**Fig. 1:**
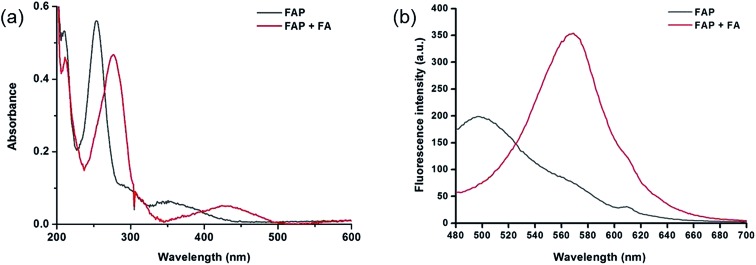
(a) UV-vis absorption spectra of **FAP** (20 μM) in the absence or presence of FA (1 mM) in PBS buffer (10 mM, pH 7.4, containing 1% MeCN) at 37 °C for 120 min. (b) Fluorescence spectra of **FAP** (20 μM) in the absence or presence of FA (1 mM) in PBS buffer (10 mM, pH 7.4, containing 1% MeCN) at 37 °C for 120 min, with excitation at 405 nm, and excitation and emission slit widths = 5 nm.

As expected, the homoallylamino modification on **FAP** leads to relatively short absorbance and fluorescence. The free **FAP** (20 μM) exhibited two absorption peaks at 254 nm and 353 nm, and one broad emission peak at 495 nm. However, the incubation of 20 μM **FAP** with 1 mM FA resulted in a remarkable red shift of the absorbance signals (23 nm and 76 nm, respectively). As shown in Fig. 1a, two obvious absorption bands with peaks at 277 nm and 429 nm newly emerged, and the intensities of the original peaks at 254 nm and 353 nm simultaneously decreased; meanwhile, accompanying the reaction of **FAP** with FA, the emission wavelength was also obviously shifted (75 nm), affording an important enhancement of the fluorescence emission band at 570 nm and a significant decrease at 495 nm (Fig. 1b). These *in vitro* responses of **FAP** validate the previous design strategy and confirm that **FAP** is a good sensor for *in vitro* formaldehyde determination. In order to elaborate the reaction mechanism, we then monitored the reactions between **FAP** and FA using HPLC and analysed the reactions using MS and IR spectroscopy. The reactions with different incubation times showed clean conversion, and a new peak (*m*/*z* = 201.1), identified as the product with the expected mass of the aromatic aldehyde (**3**), began to appear after 1 h of incubation (Fig. S1 and S2†). The reactions of different FA concentrations also showed a gradual decrease in the probe peak and a corresponding increase in the product peak with increasing FA concentration (Fig. S2†). In addition, a signal of the freshly formed –CHO– group was also clearly observed in the IR spectrum after treatment with FA (Fig. S3†). Taken together, these findings show that the formation of the aldehyde is indeed promoted by reacting with FA.

To assess the response time of **FAP** towards FA, a solution of **FAP** (20 μM) was added to FA (2 mM) in PBS buffer (10 mM, pH 7.4, containing 1% MeCN) for different incubation times and then measured by a fluorescence spectrometer (Fig. S4†). Importantly, a significant ratiometric change was observed even after 5 min, accompanying a pronounced enhancement of the fluorescence intensity at 570 nm and a distinct reduction at 495 nm, whereas the emission band at 570 nm was gradually enhanced with the extension of the incubation time even after 180 min. Specifically, the fluorescence ratio *I*_570_/*I*_495_ increased by 9-fold upon reaction with FA for 120 min. In order to observe the relatively obvious fluorescence changes at an appropriate incubation time, we chose a reaction time of 120 min to further investigate the pH effect on the probe, **FAP**. We measured the fluorescence signal ratios of *I*_570_/*I*_495_ towards FA (2 mM) in PBS buffer with various pHs ranging from 3.0 to 11.0 and, surprisingly, found that **FAP** shows a relatively wide range of pH application (Fig. S5†). Importantly, the remarkable ratiometric fluorescence response and the wide range of pH application of **FAP** are advantageous to minimizing the interference of the fluorescence background and increasing the fidelity of the detection signal.

**Fig. 2:**
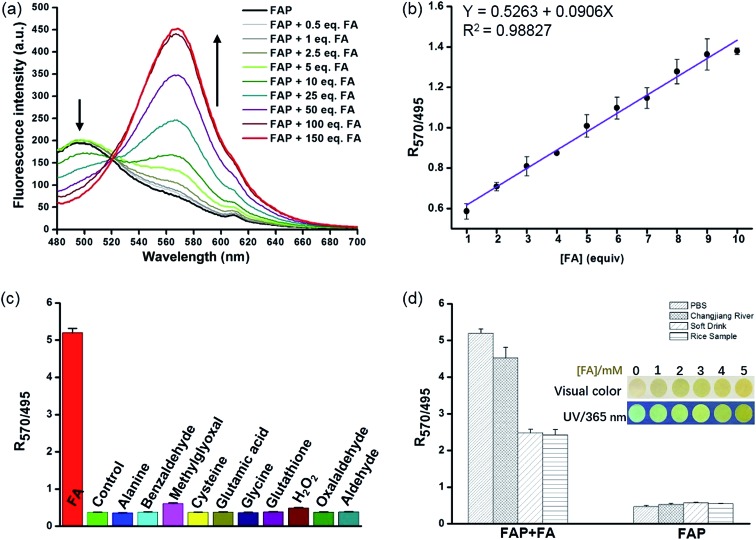
(a) Fluorescence spectra of **FAP** (20 μM) in PBS buffer (10 mM, pH 7.4, containing 1% MeCN) after treatment with FA (0–3 mM) for 120 min. (b) The linear relationship between the fluorescence intensity ratio (*I*_570_/*I*_495_) and the concentration of FA (0–10 equiv.) in PBS buffer (10 mM, pH 7.4, containing 1% MeCN) for 120 min. (c) Fluorescence intensity ratios (*I*_570_/*I*_495_) of the probe, **FAP** (20 μM), in the presence of various relevant analytes (100 equiv.) in PBS buffer (10 mM, pH 7.4, containing 1% MeCN) at 37 °C. All the fluorescence data were observed after 120 min. *λ*_ex_ = 405 nm, slit: 5 nm/5 nm. (d) Fluorescence intensity ratio (*I*_570_/*I*_495_) changes of **FAP** (20 μM) upon the addition of FA (2 mM) in different buffer systems, including PBS, Changjiang River, soft drink, and rice water samples. Inset: the visual colors or visual fluorescence colors of the filter paper strips loaded with **FAP** in the presence of different concentrations of FA (0–5 mM). All fluorescent images were captured under irradiation by a 365 nm UV lamp.

Encouraged by these results, we sought to investigate the sensitivity of **FAP** for FA, and we assessed the fluorescence response of **FAP** (20 μM) with different concentrations of FA (0–3 mM) in PBS buffer at 37 °C. The incubation of **FAP** with increasing concentrations of FA resulted in a gradual increase of the emission intensity at 570 nm and a corresponding decrease at 495 nm (Fig. 2 and S6†). Strikingly, an excellent linear dependence (*R* ^2^ = 0.98827) between the fluorescence signal ratios of *I*_570_/*I*_495_ and the concentrations of FA (0–200 μM) was observed. Moreover, to further evaluate the validity and stability of the slope, different concentrations of FA (1.5, 4.5 and 9.5 equivalent) were spiked into the PBS buffer and incubated with **FAP** (20 μM) for 120 min (Fig. S7†). After a calculation using a calibration curve, seen in Fig. 2b, the concentration of formaldehyde was determined, and the resultant FA recoveries ranged from 98% to 106%, revealing that **FAP** has potential capability for quantitatively monitoring FA. The limit of detection (LOD) of **FAP** for the detection of FA was 0.5 μM in aqueous solution based on the signal-to-noise ratio (S/N = 3).

The selectivity of the fluorescent probe is an important parameter for assessing if it is suitable for biological application. To determine whether **FAP** could detect FA specifically, we then examined the fluorescence signal and compared the ratio values of the fluorescence intensity at 570 nm to 495 nm (*I*_570_/*I*_495_) after incubation with a series of comparative analytes, including benzaldehyde, methylglyoxal, oxalaldehyde, GSH, H_2_O_2_, amino acids, *etc.* As seen in Fig. 2c and S8,† the largest change of the fluorescence signal ratio was observed upon the addition of FA, while the addition of 100 equiv. of the other analytes displayed a negligible response. **FAP** displayed a 9–13-fold greater response toward FA than the other analytes (Fig. 2c). Additionally, exposing **FAP** to a mixture of FA and other species still yielded a significantly increased fluorescence ratio (*I*_570_/*I*_495_) (Fig. S9†). These results clearly indicate that **FAP** exhibits excellent selectivity for FA over other analytes. Collectively, these selective and sensitive assays unambiguously suggest that **FAP** has potential capability for tracking FA in environmental and biological samples.

Having established **FAP** determination, we next moved on to evaluate the practicability of **FAP**. We initially carried out experiments using real-water samples from the Changjiang River, as well as soft drink and rice water samples. FA (2 mM) was previously spiked into these real-water samples and incubated with **FAP** (20 μM) for 2 hours (Fig. 2d). Obvious changes of the fluorescence signal ratio were observed after incubation of FA in these buffer systems, while the soft drink and rice water samples only showed relatively moderate changes that might be because FA was partially consumed *via* the interaction with biomolecules. But fortunately, these changes of the fluorescence signal ratio were enough to help us achieve the determination of FA in the real-water samples. Moreover, we prepared dried FA test papers by dipping paper into a **FAP** solution and further evaporating to remove the solvent (Fig. 2d inset). Interestingly, using this simple FA test paper to detect FA solutions with various concentrations exhibited quick color changes from green to yellow within several seconds, indicating the potential utility of **FAP** as a simple sensor for tracking FA in environmental samples.

**Fig. 3:**
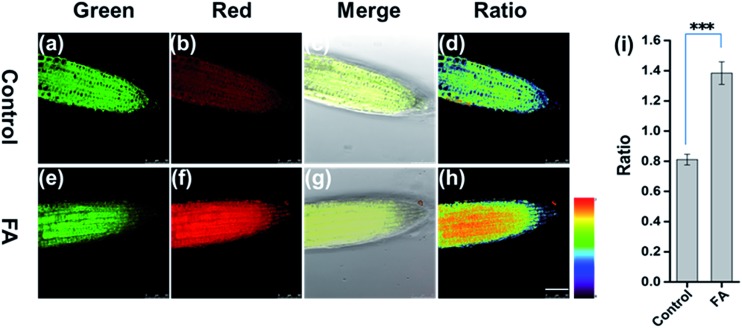
Confocal microscopy images of *Arabidopsis thaliana* incubated with **FAP** (50 μM) for 45 min at 37 °C (a–d), followed by the addition of 3 mM HCHO (e–h) and incubation for another 2 hours. (a and e) The green channel (475–520 nm), (b and f) the red channel (545–595 nm), (c and g) the merge images of the bright-light field, green channel and red channel, and (d and h) the ratio images of the red to green channel. *λ*_ex_ = 405 nm, scale bar = 50 μm. (i) Quantification of the imaging data (d and h). *n* = 3, error bars were ±SD. Statistical analyses performed with a two-tailed Student’s *t*-test with unequal variance, ***p value < 0.0001.

To address the capability of **FAP** for mapping FA in live tissue, we employed **FAP** to image FA in the live root tip tissue of *Arabidopsis thaliana* using a confocal fluorescence microscope. As shown in Fig. 3, the root tip tissues of *Arabidopsis thaliana* were pretreated with **FAP** (50 μM) for 45 min, and subsequently different concentrations of exogenous FA (0 or 3 mM) were added and incubated for another 120 min. Gratifyingly, **FAP** can effectively penetrate the cell wall and cell membrane, and can display a bright fluorescence in the green channel and relatively weak fluorescence in the red channel, suggesting that live *Arabidopsis thaliana* tissues have a low level of intracellular FA activity.^12,13^ Importantly, upon incubation with a typical stress concentration of exogenous FA (3 mM), a significant change of the fluorescence signal ratio is observed in the ratiometric fluorescence image (*F*_red_/*F*_green_) (Fig. 3). This indicates that the alteration of the intracellular FA levels in live plant tissues can be reflected by the fluorescence change of **FAP**. Notably, these FA concentrations fall well within the scope of physiological concentration, treating the condition of formaldehyde stress in plants, and are estimated to be 0.1–10 μmol g^–1^ in the fresh weight of a plant,^13^ and up to 2–8 mM in the external treatment of HCHO stress.^14,15,37^

**Fig. 4:**
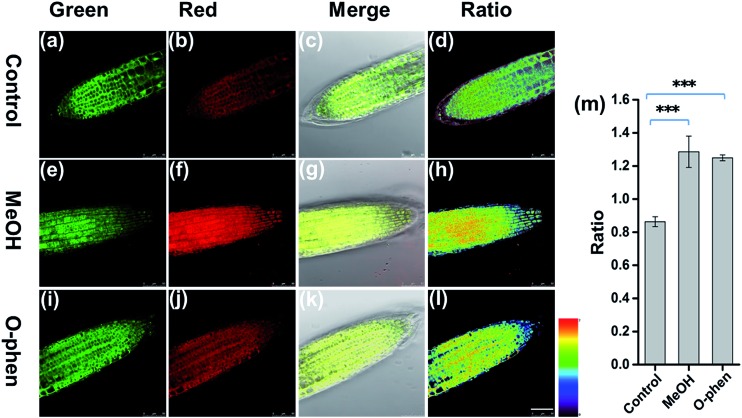
Confocal microscopy images of endogenous FA in *Arabidopsis thaliana via* pretreatment with or without 10% MeOH and 1 mM *o*-phenanthroline in PBS buffer for 1 h, followed by exchange into fresh PBS and further incubation with **FAP** (50 μM) for 3 h at 37 °C before imaging. (a, e, and i) Green channel (475–520 nm), (b, f, and j) red channel (545–595 nm), and (c, g, and k) merge images of the bright-light field, green channel and red channel, and (d, h, l) the ratio images of the red to green channel. *λ*_ex_ = 405 nm, Scale bar = 50 μm. (m) Quantification of the imaging data (d, h, l). *n* = 3, the error bars were ±SD. Statistical analyses performed with a two-tailed Student’s *t*-test with unequal variance, ***p value < 0.001.

To further investigate whether **FAP** can be applied to detect the change of endogenous FA levels in live plant tissues, we pretreated live root tip tissues of *Arabidopsis thaliana* with 10% MeOH stress for 1 h to induce the production of endogenous FA,^38^ then incubated with **FAP** (50 μM) for another 3 hours and imaged after washing with PBS (Fig. 4). The endogenous FA freshly formed *via* MeOH pretreatment results in an obvious change of the fluorescence signal ratio in the ratiometric fluorescence image compared with the negative control. Furthermore, the stimulation of endogenous FA uptake with 1 mM *o*-phenanthroline, a known inhibitor of formaldehyde dehydrogenase,^39^ also yielded a statistically significant increase in the fluorescence ratio (Fig. 4). All of these data suggest that **FAP** is capable of ratiometric monitoring of endogenous FA activity in *Arabidopsis thaliana in vivo*. Finally, we tried to apply **FAP** to measure the endogenous formaldehyde concentration in the fresh root and leaf tissues of *Arabidopsis thaliana*. Spiked HCHO was used as the internal standard in the prepared tissue homogenate. The spiked samples were subsequently incubated with the 20 μM probe for 2 hours and then monitored (Fig. S10†). We found that the concentrations of endogenous formaldehyde in the fresh homogenates of the *Arabidopsis thaliana* root and leaf tissues are 1.8 μmol g^–1^ and 4.2 μmol g^–1^, respectively. This result is consistent with the previous report that the average endogenous FA concentration was estimated to be 0.1–10 μmol g^–1^ of the fresh weight of the plant.^13^ Taken together, these findings demonstrate that **FAP** displays the ability of detection of the biologically relevant endogenous formaldehyde in live plant tissues.

## Conclusions

In summary, we have successfully developed a ratiometric fluorescent probe, **FAP**, for monitoring formaldehyde based on the *N*,*N*-dimethylquinolin-6-amine fluorophore for the first time. This probe displayed a significant red shift (75 nm) in the emission profiles and an obvious change of the fluorescence signal ratio for the response of FA. We have demonstrated that this highly selective and effective probe has potential capability for monitoring FA in real-water samples and live plant tissues. *In vivo* imaging results show that **FAP** can not only effectively map exogenous FA, but also can sensitively track endogenous FA. To the best of our knowledge, this is the first example of a fluorescent probe-based methodology for the ratiometric imaging of endogenous FA in plant tissues. With these insights, we anticipate that the further application of **FAP** to the fluorescent imaging of FA in other plants will enable better understanding of its metabolism mechanism, thus promoting the progress of FA related physiological and pathological studies.
