# Supplementary material for: Imaging of formaldehyde in plants with a ratiometric fluorescent probe
Source: Chem Sci. 2017 Jun 6;8(8):5616–21. doi: 10.1039/c7sc00373k (PMC5621015; doi:10.1039/c7sc00373k)
Supplement: Supplementary file 1 [file SC-008-C7SC00373K-s001.pdf]

Electronic Supplementary Material (ESI) for Chemical Science.
This journal is © The Royal Society of Chemistry 2017

Supplementary Information

Imaging of formaldehyde in plant with a ratiometric fluorescent probe

Zhen Li[a†], Yuqing Xu[a†], Hailiang Zhu[a,*], Yong Qian[a,b,*]

[a]*State Key Laboratory of Pharmaceutical Biotechnology, School of Life Sciences, Nanjing University, No.163 Xianlin Road, Nanjing 210023, China. E-mail: yongqian@nju.edu.cn*

[b]*College of Chemistry and Materials Science, Nanjing Normal University, No.1 Wenyuan Road, Nanjing, 210046, China. E-mail: yongqian@njnu.edu.cn*

†*These authors contributed equally to this work.*

Table of Content

1. *Materials and equipment..........S3*
2. *Synthesis of compounds..........S3-S5*
3. *Determination of the fluorescence quantum yield..........S5*
4. *The limit of detection (LOD) of FAP..........S5*
5. *FA detection with FAP in real water samples..........S5*
6. *Determination of FA with a simple FA testing paper..........S6*
7. *Arabidopsis Thaliana culture and imaging..........S6-S7*
8. *Supplementary Figures..........S7-S15*
9. *^{1}H and ^{13}C NMR spectra..........S16-S18*

1. Materials and equipment

All chemicals were available commercially and used without further treatment. Chromatographic purification of products was performed on silica gel (300-400 mesh). Buffer solution (PBS 1×, pH 7.4) was prepared using deionized water. The imaging experiments were carried out using two-photon confocal fluorescent microscope (Leica TCS SP8 MP, Nanjing University). UV absorption spectra were accomplished on UV spectrometer. All fluorescence measurements were recorded on Hitachi Fluorescence Spectrophotometer F-7000. ^{1}H NMR and ^{13}C NMR spectra were acquired in the solvent stated using a Bruker DRX-400 (400 MHz). Mass spectra were obtained by Mass Spectrometry Facility at Nanjing University. The type of fourier transform infrared spectrometer was NEXUS870.

Formaldehyde standard solution (10 mg/ml) was firstly diluted into a stock solution (10 mM) for further testing, other analytes inculding Alanine, Benzaldehyde, Methylglyoxal, Cysteine, Glutamic acid, Glycine, Glutathione, H_2O_2, Oxalaldehyde, Aldehyde were dissolved in ddH_2O to obtain 10 mM stock solution, seperaterly. Probe **FAP** was dissolved in MeCN to get a 1 mM stock solution. For all measurements, the excitation wavelength was 405 nm, and both excitation and emission slit widths were 5 nm. After incubation with the various analytes for 2 h at 37 ℃, the emission spectrum was measured and scanned from 415 nm to 700 nm at 1200 nm/min.

2. Synthesis of compounds

2.1. Synthesis of N,N,2-trimethylquinolin-6-amine (2)

4-*N,N*-Dimethylamino aniline (**1**) (5 g, 36.7 mmol, 1.0 equiv) was dissolved in a solution of HCl (6 M, 66 mL), after addition of crotonaldehyde (6.0 mL, 73.5 mmol, 2 equiv), the result mixture was stirred at room temperature for 1 h. Then toluene (35 mL) was added and the reaction was further refluxed at 115 ℃ for overnight. After cooling down to room temperature, the toluene layer was removed and saturated sodium hydroxide solution was added to neutralize the aqueous layer. The solution was extracted with dichloromethane, washed twice with saturated NaCl solution, and dried over anhydrous sodium sulfate, filtered and concentrated under

reduced pressure. The crude product was purified by column chromatography on silica gel (PE: EA = 4:1 v/v) to obtain **2** as a sand-brown solid (3.6 g, 70%). ^{1}H NMR (400 MHz, $CDCl_3$) δ 7.92 (d, *J* = 9.3 Hz, 1H), 7.89 (d, *J* = 8.5 Hz, 1H), 7.37 (d, *J* = 2.8 Hz, 1H), 7.19 (d, *J* = 8.4 Hz, 1H), 6.81 (d, *J* = 2.8 Hz, 1H), 3.07 (s, 6H), 2.70 (s, 3H). ^{13}C NMR (100 MHz, $CDCl_3$) δ (ppm) 154.46, 148.22, 141.60, 134.68, 128.91, 127.82, 122.16, 119.45, 105.39, 40.83, 24.78.

2.2. *Synthesis of 6-(Dimethylamino) quinoline-2-carbaldehyde (3)*

A mixture of selenium dioxide (2.8 g, 25.1 mmol, 1.3 equiv) in dioxane/water (140 mL/14 mL) was heated at 80 ℃ for 30 min, compound **2** (3.5 g, 19.2 mmol, 1 equiv) was then added and the mixture was stirred at 80 ℃ for 4 h. After cooled down to room temperature, the mixture was filtered through diatomite, then the filter residue was flushed many times by a small amount of methylene chloride. The filtrate was concentrated under reduced pressure to afford crude product that purified by column chromatography on silica gel (PE: EA = 6:1 v/v) to afford the corresponding aldehyde **3** as a yellow solid (1.52 g, 40%). ^{1}H NMR (400 MHz, $CDCl_3$) δ 10.14 (s, 1H), 8.07 (d, *J* = 9.4 Hz, 1H), 8.02 (d, *J* = 8.5 Hz, 1H), 7.92 (d, *J* = 8.5 Hz, 1H), 7.43 (dd, *J* = 9.4, 2.7 Hz, 1H), 6.80 (d, *J* = 2.6 Hz, 1H), 3.17 (s, 6H). ^{13}C NMR (100 MHz, $CDCl_3$) δ (ppm) 193.48, 150.20, 148.87, 141.70, 134.25, 132.24, 131.29, 119.73, 118.13, 103.86, 40.41.

2.3. Synthesis of 2-(1-aminobut-3-en-1-yl)-N, N-dimethylquinolin-6-amine (FAP)

Potassium allyltrifluoroborate (444 mg, 3 mmol) was dissolved in a 7 N solution of NH_3 in MeOH (4 mL) and stirred at room temperature for 15 min. Dissolved compound **3** (300 mg, 1.5 mmol) in 7 N solution of NH_3 in MeOH (3 mL), and then the solution was added to the above reaction which further was stirred for 16 h. The reaction was poured into saturated $NaHCO_3$ (100 mL), extracted with EtOAc and the extraction solution was dried with anhydrous sodium sulfate and concentrated under reduced pressure. The crude residue was purified by silica gel chromatography using DCM/MeOH (20:1, v/v) as eluent to yield probe **FAP** as a light yellow oil (78 mg, 22%). ^{1}H NMR (600

MHz, $CDCl_3$) 7.83 (d, 1H, *J* = 3.2 Hz), 7.82 (d, 1H, *J* = 3.6 Hz)，7.26 (dd, 1H, J_1 = 9.3 Hz, J_2 = 2.6 Hz)，7.23 (d, 1H, *J* = 4.3 Hz)，6.71 (d, 1H, *J* = 1.2 Hz)，5.72 (m, 1H), 5.03 (dd, 1H, J_1 = 30.7 Hz, J_2 = 13.7 Hz), 4.13 (dd, 1H, J_1 = 7.9 Hz, J_2 = 5.3 Hz), 2.97 (s, 6H), 2.58 (m, 1H), 2.38 (m, 2H), ^{13}C NMR ($CDCl_3$, 150 MHz) δ (ppm): 159.6, 148.4, 141.5, 135.2, 134.7, 129.6, 128.8, 119.5, 119.4, 117.9, 105.3, 56.7, 43.0, 40.8. HR MS calculated for $C_{15}H_{20}N_3$ $[M+H]^+$ m/z 242.16572, found 242.16562.

3. Determination of the fluorescence quantum yield

The fluorescence quantum yield Φ_u was estimated from the absorption and fluorescence spectra of FAP according to equation, where using the ethanol solution of rhodamine B (10 μM , Φ = 0.69, λex = 365 nm) for the sample and reference. The fluorescence quantum yield was calculated using equation as follows:

$\Phi_u = [(A_s F_u n^2)/ (A_u F_s n_0^2)]\Phi_s$.

Φ_s is the quantum yields of reference substance , A_s and A_u represents the absorbance of the reference and testing solution at the excitation wavelength and controlled to be lower than 0.05 at the same time, F_s and F_u refer to the integrated emission band areas, n and n_0 are the solvent refractive indexes of sample and reference, respectively.

Quantum yield: Φ = 0.72.

4. The limit of detection (LOD) of FAP

The emission spectrum of free FAP in PBS buffer (10 mM, pH = 7.40, containing 1% MeCN) was collected for 20 times to confirm the background noise σ. The linear regression curve was then fitted according to the data in the range of FA from 0 to 10 equiv and obtained the slope of the curve. The detection limit (3σ/slope) was then determined to be 0.50 *μ*M, which facilitate the quantitative detection of FA in complex environment.

5. FA detection with FAP in real water samples

FA (2 mM) was previously spiked into the real-water samples: PBS buffer (10 mM, pH 7.4), Changjiang River, Soft Drink, Rice sample buffer (200 mg/5 mL,

1.0% MeCN), and then incubated with FAP (20 μM) for 2 hours at 37 ℃, the fluorescence intensity were measured and their ratio (I_{570}/I_{495}) were calculated. The results demonstrats FAP is suitable for the detection of FA in real-environmental samples.

6. Determination of FA with a simple FA testing paper

Circular filter paper was cut out of uniform, then soaked in FAP (50 μM) solution and further evaporated to remove the solvent. Different concentration of formaldehyde (0–5 mM) solution was dropwisely added to the FA testing paper and reacted several seconds. The visual colors or visual fluorescence colors of testing paper were imaged in visible light and irradiation by a 365 nm UV lamp, respectively.

7. Arabidopsis Thaliana culture and imaging

Arabidopsis Thaliana used the wild type, and seeds were dipped in 1 mL of 75% alcohol for 10 min. The seeds were cultivated in 50% basal medium with vitamins (MS) and contained 1% sucrose, 1% agar at the same time. Set the culture medium with potassium hydroxide as pH 5.8. It was encased with tin foil, and then placed in the refrigerator at 4°C for two days. Removed the tin foil, transferred into the incubator and further incubated for 5 days. The 5-days-old *Arabidopsis Thaliana* was selected for next experiments.

The 5-days-old *Arabidopsis Thaliana* were incubated with 50 μM FAP in PBS buffer for 45 min at 37℃. After washing with PBS buffer to wipe out the residual probe in the surface, the Arabidopsis Thaliana were further incubated with 3 mM FA for 2 h at 37°C. The *Arabidopsis Thaliana* with or without FA incubation were imaged respectively by two-photon confocal microscope. For monitoring the endogenous FA, *Arabidopsis Thaliana* was pretreated with 10% MeOH or 1mM o-phenanthroline in PBS buffer to induce the production or uptake of endogenous FA at 37°C for 1 h, then incubated with FAP (50 μM) for another 3 hours and imaged after washing with PBS three times.

To monitor the endogenous formaldehyde in homogenate, we choose the root and leaf tissues of 10 days-old *Arabidopsis Thaliana*. The tissues were grinded in 1X PBS (1 g/mL), then centrifuged with 8000 r/min for 15 min, and

filtrated through membrane to get the transparent tissue homogenate. After a 2 times dilution, the reaction system was set as 1 μL sample, 2 μL FAP (20 μM) in 197 μL PBS buffer (10 mM, pH 7.4, 1.0% MeCN). Five points were set as 0, X, X+1, X+2 and X+3. 1 mM $NaHSO_3$ was added into Point 0, while 20 μM, 40 μM and 60 μM FA were spiked into Point X+1, X+2 and X+3 respectively. The reactions were incubated at 37 ℃ for 2 hours before measured by a fluorescence spectrometer.

8.Supplementary Figures

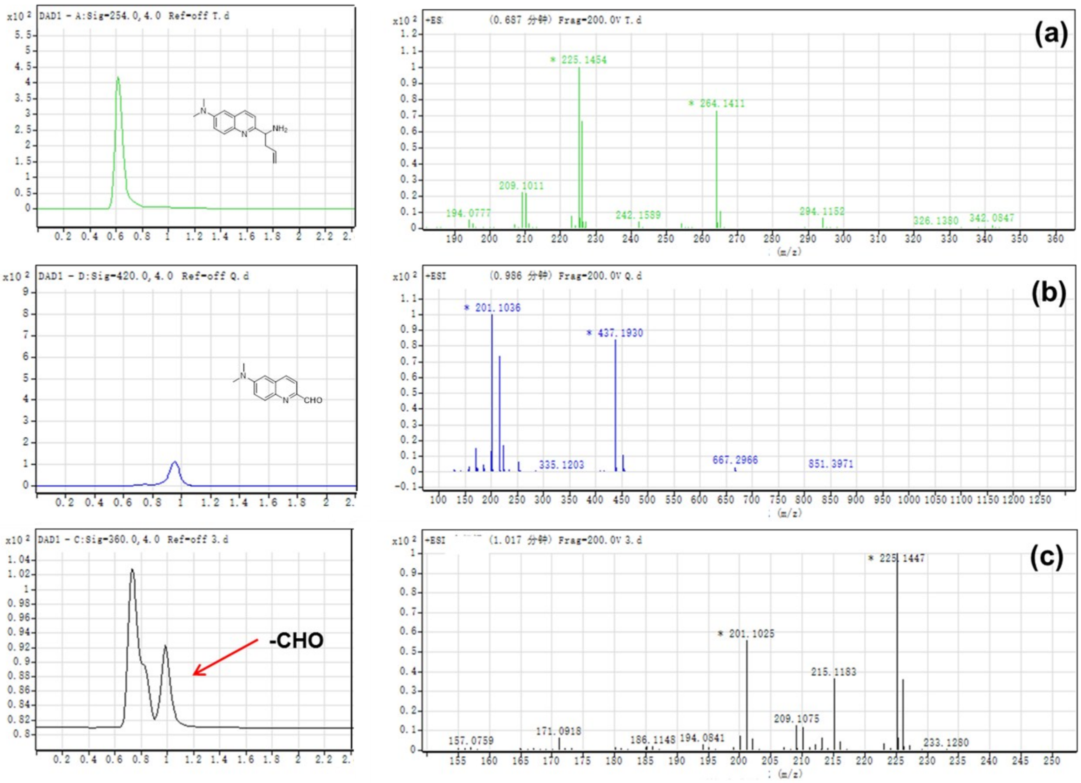

Figure S1. **(a)** LC-MS spectrum of compound **FAP** (calculated for $C_{15}H_{20}N_3$ $[M+H]^+$ 242.1657, found 242.1589; calculated for $C_{15}H_{19}N_3Na$ [M+Na]+ 264.1477, found 264.1411). **(b)** LC-MS spectrum of compound (3) (calculated for $C_{12}H_{13}N_2O$ $[M+H]^+$ 201.1028, found 201.1036). **(c)** LC-MS of **FAP** solution after incubation with FA. A new peak (m/z = 201.1) identified as the aromatic aldehyde (3) (calculated for $C_{12}H_{13}N_2O$ $[M+H]^+$ 201.1028, found 201.1025). LC-MS was conducted using Agilent 6530 Accurate-Mass Q-TOF LC/MS with Agilent EC-C18 column, 2.7 μm, 4.6*50 mm. HPLC runs used a linear gradient from 70% H_2O/30% CH_3OH to 0% H_2O/100% CH_3OH over 6 min and

then an extra 3 min with H_2O/100% CH_3OH. The current velocity is set at 0.4 mL/min.

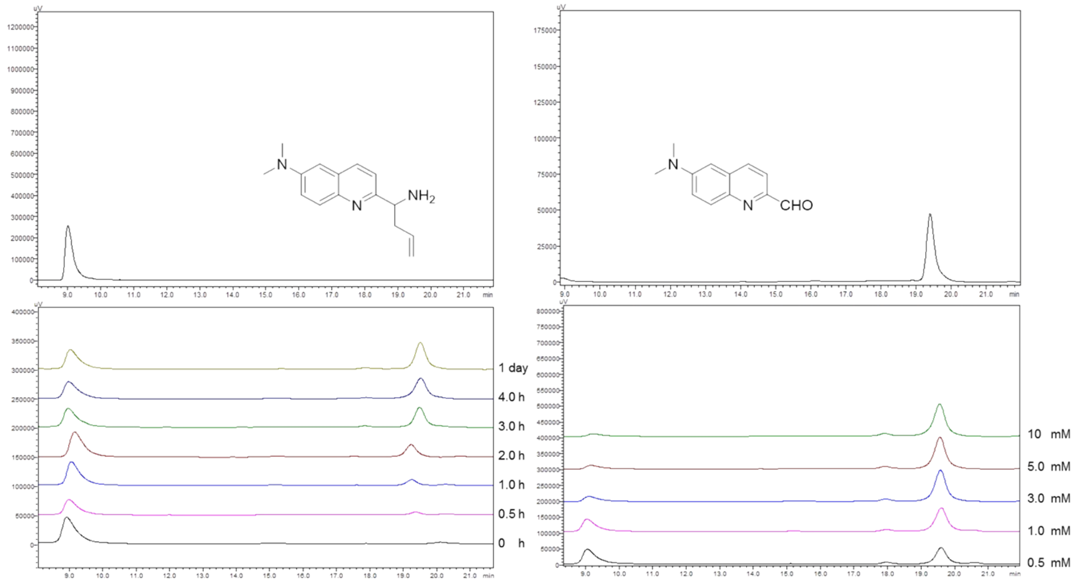

Figure S2. **(a)** HPLC analysis of 100 μM **FAP** in acetonitrile solution. **(b)** HPLC analysis of 100 μM compound (**3**) in acetonitrile solution. **(c)** HPLC analysis of

reaction between 100 μM FAP and 3 mM FA at 37 ^{o}C in 10 mM PBS (pH 7.4). The time points are 0, 0.5, 1.0, 2.0, 3.0, 4.0, 24 h. **(d)** HPLC analysis of reaction between 100 μM FAP and different concentration of FA at 37 ^{o}C in 10 mM PBS (pH=7.4) for 4 h. Formaldehyde concentration are 0.5, 1.0, 3.0, 5.0, 10.0 mM. The experiment is conducted using Shimazu LC-20AT liquid chromatograph with Shimazu Inert Sustain C18 column, 5 μm, 4.6*250 mm. HPLC runs used a linear gradient from 30% MeCN/70% 0.5% NH_4OAc to 65% MeCN/35% 0.5%NH_4OAc over 25 min. The current velocity is set at 1.0 mL/min. Column temperature is 30 ^{o}C. UV detector wavelength is set at 260 nm.

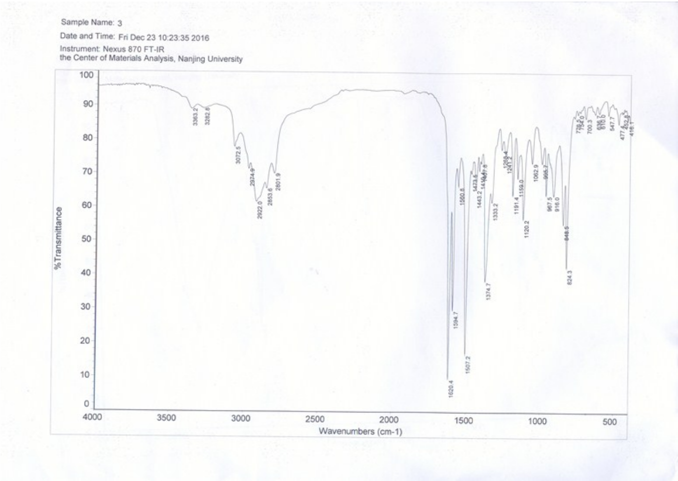

Figure S3a. IR spectra of compound **FAP**.

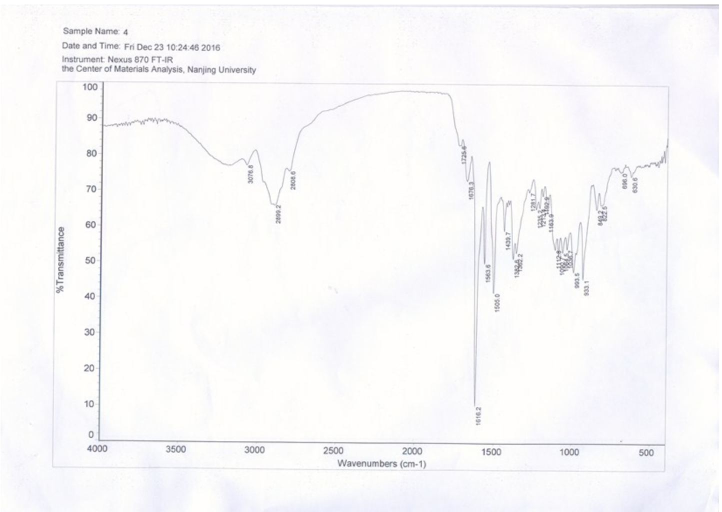

Figure S3b. IR spectra of compound **FAP+FA**.

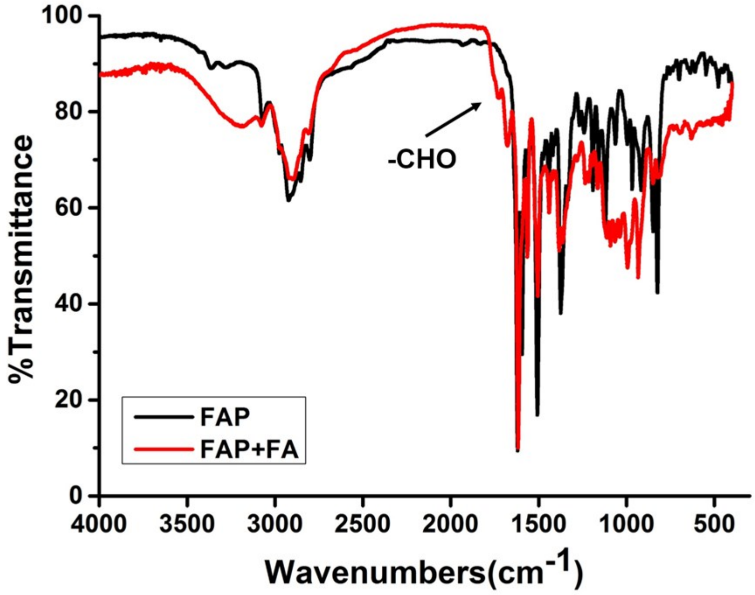

Figure S3c. IR spectras of FAP before (black) and after (red) treatment with FA. A signal of the freshly formed –CHO- group was clearly observed.

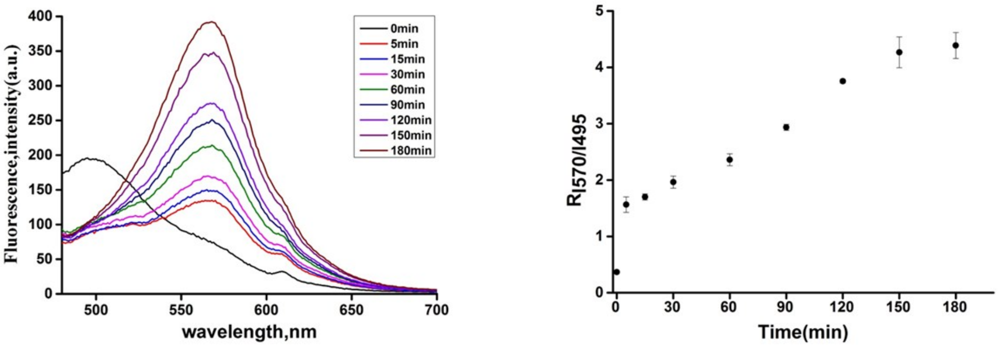

Figure S4a. (Left) Time-dependent (0 to 180 min) fluorescence spectra changes of FAP (20 μM) toward FA (2.0 mM) in 10 mM PBS buffer (pH 7.4, 1.0% MeCN) at 37 ℃. (Right) The fluorescence intensity ratio (I_{570}/I_{495}) of FAP (20.0 μM) with FA (2 mM) was recorded at the different incubation time. Time points are 0, 5, 15, 30, 60, 90, 120, 150, 180 min. λ_{ex} = 405 nm. Slit: 5 nm/5 nm. Error bars are ± SD, n = 3.

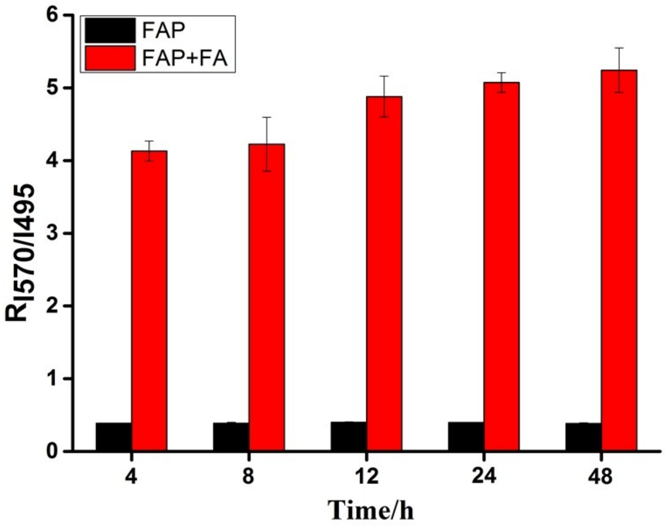

Figure S4b. Determination of the stability. Fluorescence intensity ratio (I_{570}/I_{495}) of free FAP (20.0 μM) and incubation with FA (2.0 mM) in PBS buffer (10 mM, pH 7.4, 1.0% MeCN) for different incubation time (0- 48 h). λ_{ex} = 405 nm, slit: 5 nm/5 nm. The data represents the average of three independent experiments.

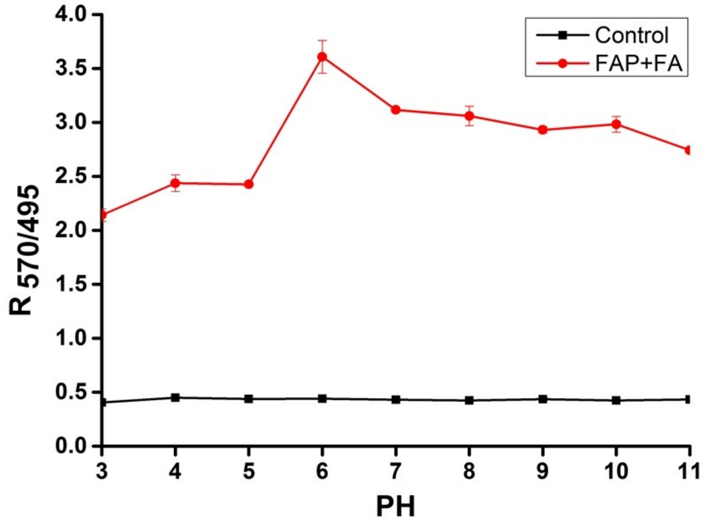

Figure S5. The changes of fluorescence intensity ratio (I_{570}/I_{495}) of FAP (20 μM) *versus* the reaction incubated with or without FA (2.0 mM) in PBS buffer with different pH values at 37 °C for 2 h. λ_{ex} = 405 nm, slit: 5 nm/5 nm. The data represents the average of three independent experiments.

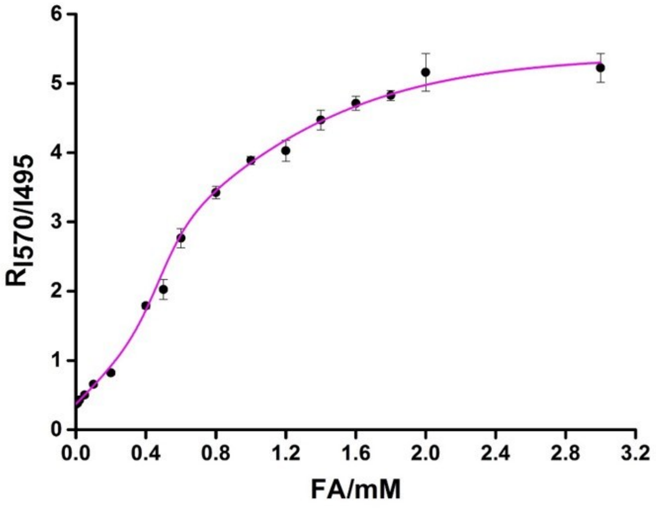

Figure S6. The fluorescence intensity ratio (I_{570}/I_{495}) was recorded of FAP (20.0 μM) with the various concentrations of FA (0 – 3.0 mM) in PBS buffer (10 mM, pH 7.4, containing 1% MeCN) after 2 h of incubation. λ_{ex} = 405 nm, slit: 5 nm/5 nm. The diagram indicated that the fluorescence intensity ratio was gradually elevated with the increasing amounts of FA, when the concentration of formaldehyde came to the 2 mM, the detection would tend to competely. The data represents the average of three independent

experiments.

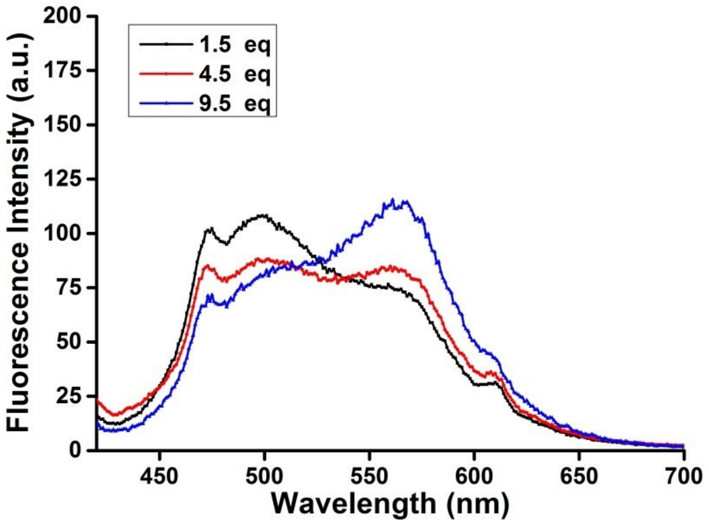

Concentration added (eq)	I_{570}/I_{495}	Concentration found (eq)	Recovery %	RSD %
1.5	0.6708	1.595	106.3	0.02322
4.5	0.9274	4.427	98.4	0.01919
9.5	1.4110	9.768	102.8	0.06245

Recovery % = 100% × (concentration found / concentration added)

Figure S7. Fluorescence spectra of FAP (20 μM) in PBS buffer (10 mM, pH 7.4, containing 1% MeCN) upon addition of 1.5, 4.5 and 9.5 eq FA respectively. Fluorescence intensity ratio (I_{570}/I_{495}) were calculated and then substituted into the linear relationship to get the recovery rate. All the fluorescence data were observed after 120 min at 37℃. λex = 405 nm, emission: 415-700 nm, slit, 5 nm/5nm, 800V.

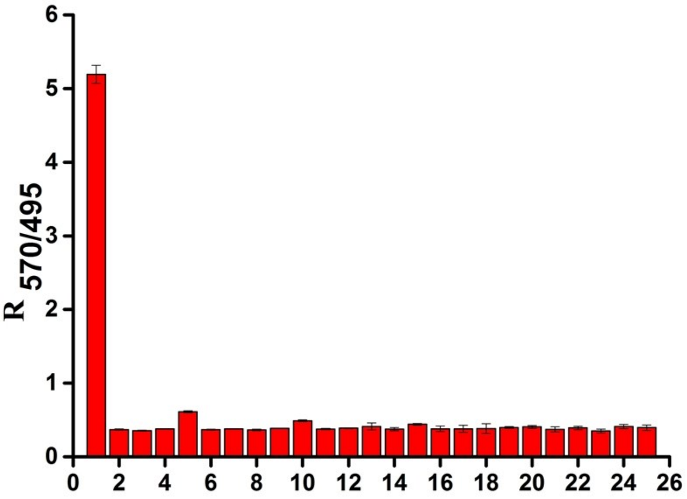

Figure S8. Fluorescence intensity ratios (I_{570}/I_{495}) of probe FAP (20 μM) in the presence of various relevant analytes (100 equiv.) in PBS buffer (10 mM, pH 7.4, containing 1% MeCN) at 37°C. All the fluorescence data were observed after 120 min. λ_{ex} = 405 nm, slit: 5 nm/5 nm. Legend: (1) FA; (2) control; (3) alanine; (4) benzaldehyde; (5) methylglyoxal; (6) cysteine; (7) glutamic acid; (8) glycine; (9) glutathione; (10) H_2O_2; (11) oxalaldehyde; (12) aldehyde; (13) arginine; (14) $CaCl_2$; (15) p-chlorobenzaldehyde; (16) Valine; (17) GSH; (18) $NaHCO_3$; (19) homocysteine; (20) $NaHSO_3$; (21) $MgCl_2$; (22) $NaNO_3$; (23) phenylalanine ; (24) Na_2S; (25) Na_2SO_3.

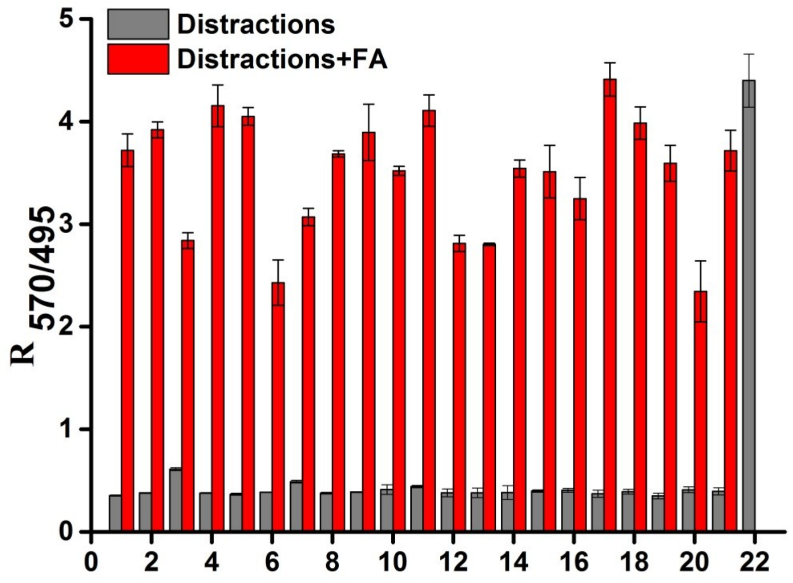

Figure S9. Competing response of FAP (20.0 *μ*M) towards various species (2.0 mM) in 10 mM PBS buffer (pH 7.4, 1.0% MeCN) at 37 ℃. The gray bars represent the addition of one of these analytes to the solution of FAP; The red bars represent the fluorescence intensity ratio (I_{570}/I_{495}) of FAP in the presence of both FA(2.0 mM) and other various analytes. All the fluorescence data were obtained after 2 h incubation. λ_{ex} = 405 nm, slit: 5 nm/5 nm. Legend: (1)Alanine, (2) Benzaldehyde, (3) Methylglyoxal, (4) Glutamic acid, (5) Glycine, (6) Glutathione, (7) H_2O_2, (8) Oxalaldehyde, (9) Aldehyde, (10) Arginine; (11) P-chlorobenzaldehyde; (12) Valine; (13) GSH; (14) $NaHCO_3$; (15) Homocysteine; (16) $NaHSO_3$; (17) $MgCl_2$; (18) $NaNO_3$; (19) Phenylalanine; (20) Na_2S; (21) Na_2SO_3; (22) FA. The data represents the average of three independent experiments.

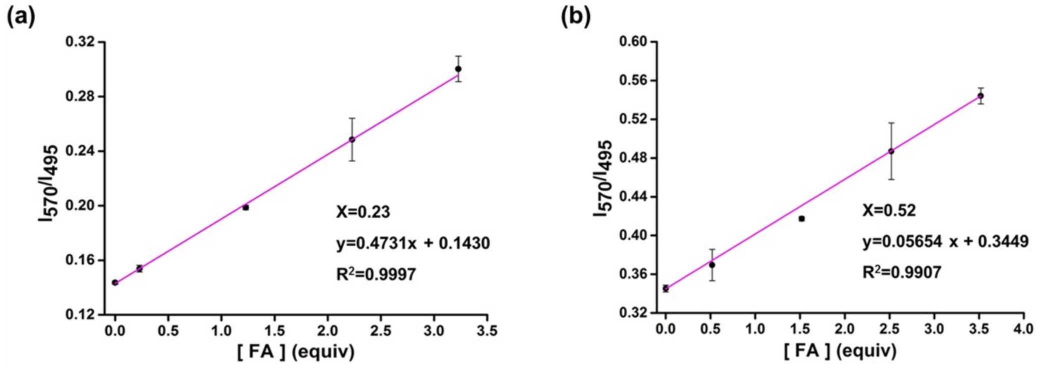

Figure S10. Determination of FA concentrations in *Arabidopsis Thaliana* root ***(a)*** and leaf ***(b)*** tissues with FAP (20 μM). Five points are set as 0 (pretreated with 1 mM $NaHSO_3$), X, X+1 (X+20 μM, spiked 20 μM FA as internal standard), X+2 (X+40 μM, spiked 20 μM FA as internal standard) and X+3 (X+60 μM, spiked 20 μM FA as internal standard). The calculated FA concentration is 0.23 eq in root tissues and 0.52 eq in leaf tissues. The FA concentrations in *Arabidopsis Thaliana* tissues were experessed as μmol/g fresh weight. The tested FA concentration in *Arabidopsis Thaliana* root and leaf tissues are calculated as 1.83 μmol/g and 4.15 μmol/g respectively, which is also consistent with the reported 0.1-10 μmol/g in references. λ_{ex} = 405 nm, slit: 5 nm/5 nm.

9.[1]*H and* [13]*C NMR spectra*

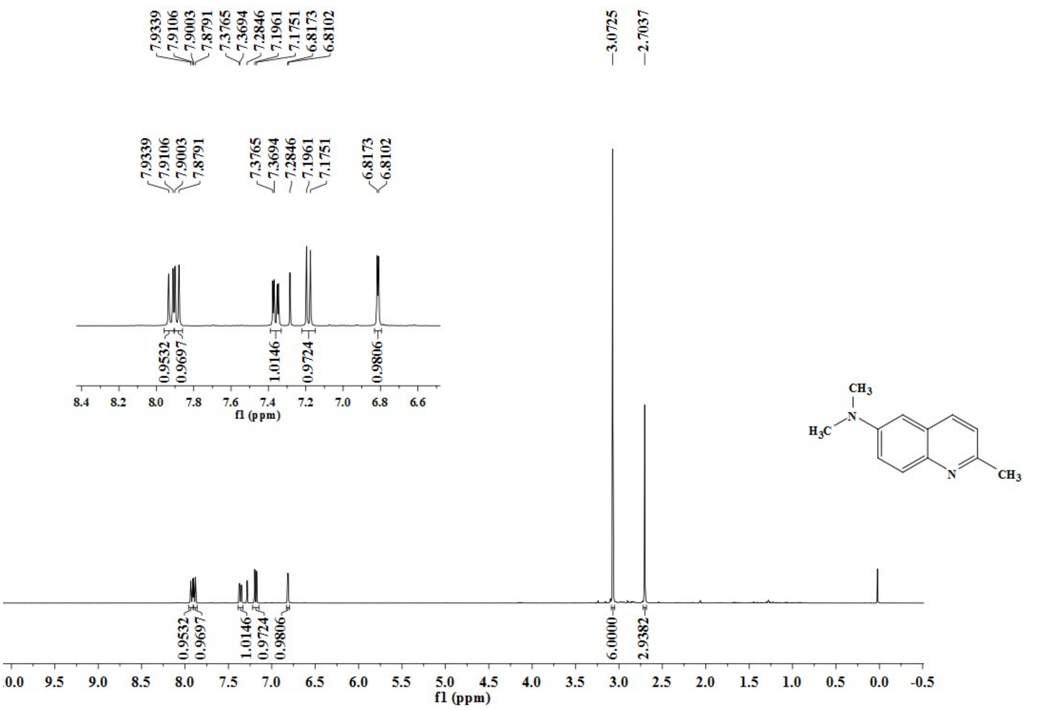

Figure S11. ^{1}H NMR of compound **2** (400 MHz, in $CDCl_3$)

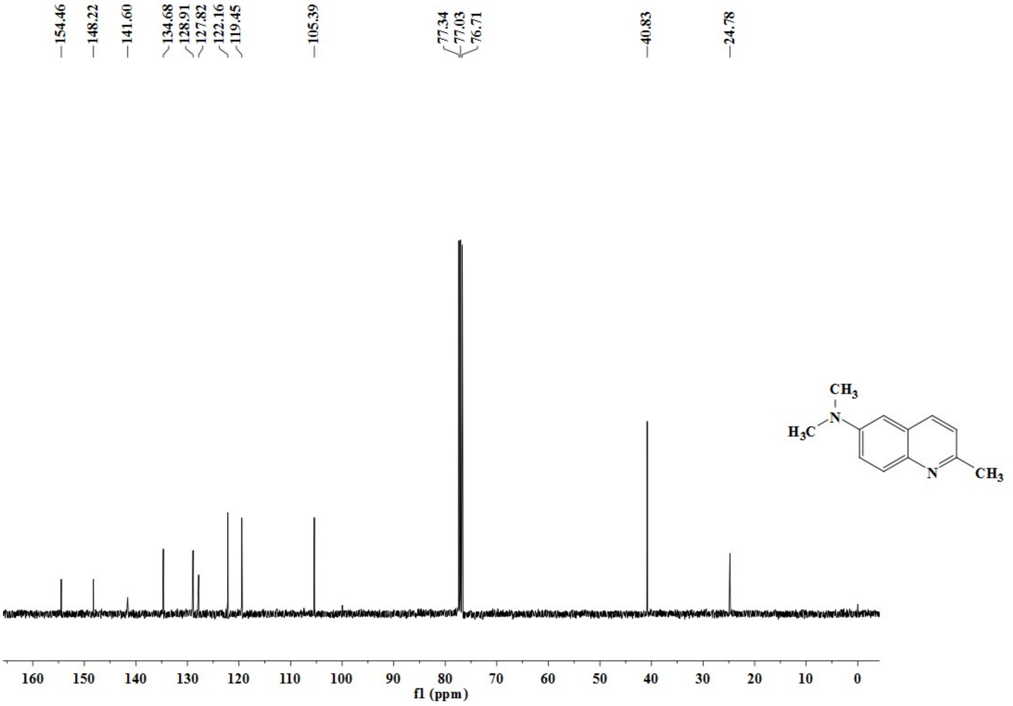

Figure S12. ^{13}C NMR of compound **2** (100 MHz, in $CDCl_3$)

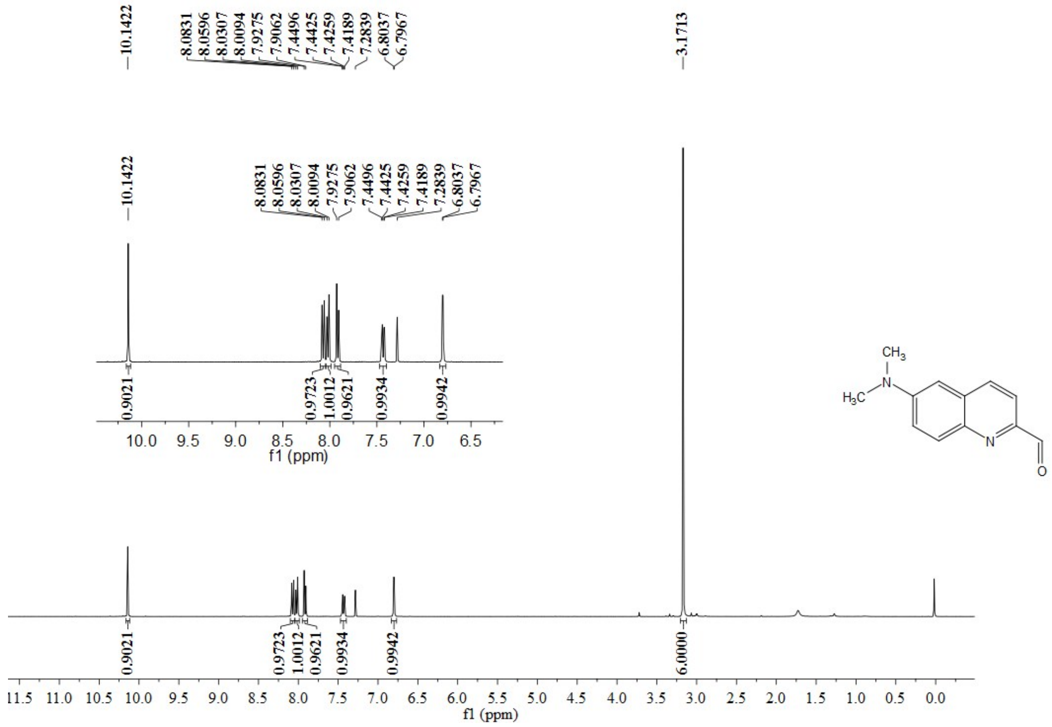

Figure S13. ^{1}H NMR of compound **3** (400 MHz, in $CDCl_3$)

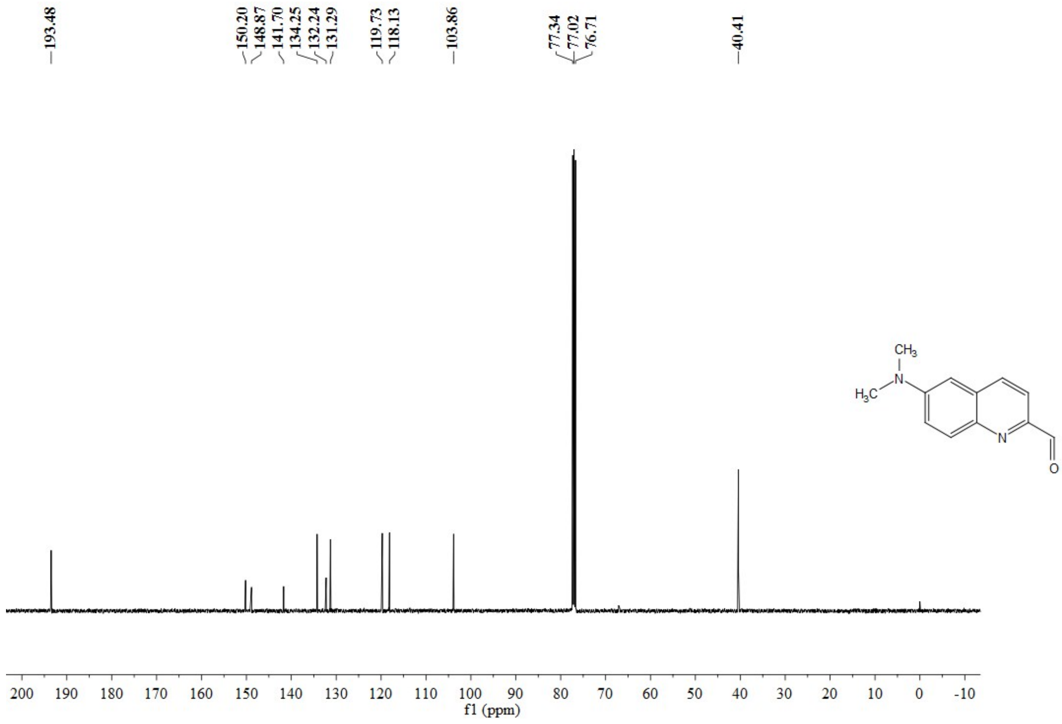

Figure S14. ^{13}C NMR of compound **3** (100 MHz, in $CDCl_3$)

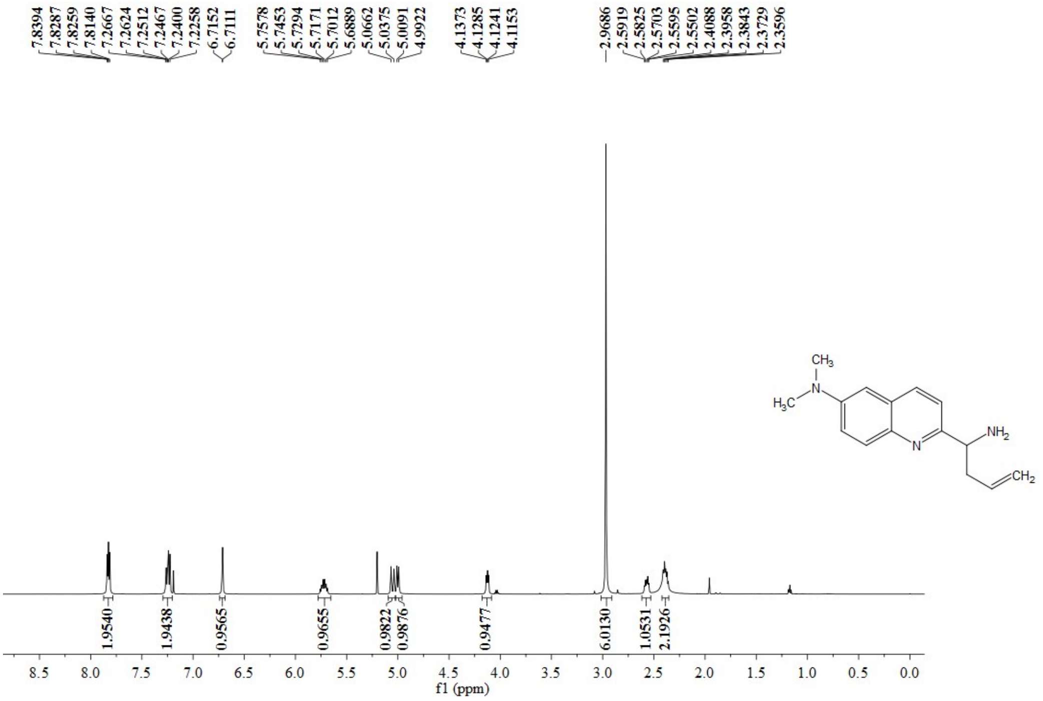

Figure S15. ^{1}H NMR of compound **FAP** (600 MHz, in $CDCl_3$)

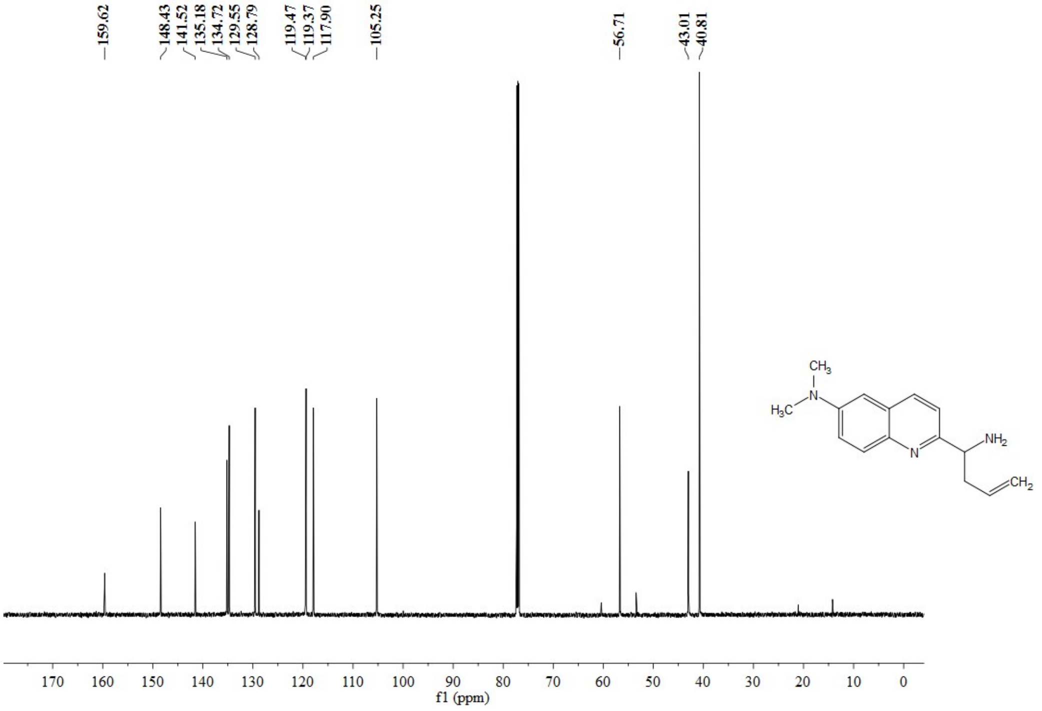

Figure S16. ^{13}C NMR of compound **FAP** (150 MHz, in $CDCl_3$)
